# Extraction of intra-biliary hepatocellular carcinoma by endoscopic retrograde cholangiopancreatography

**DOI:** 10.1186/s12876-020-01552-0

**Published:** 2020-12-07

**Authors:** Chieh Sian Koo, Khek Yu Ho, Yin Huei Pang, Daniel Q. Huang

**Affiliations:** 1 Division of Gastroenterology and Hepatology, National University Centre for Organ Transplantation, National University Hospital, Singapore, Singapore; 2 Department of Medicine, Yong Loo Lin School of Medicine, National University of Singapore, Singapore, Singapore; 3 Department of Pathology, National University Hospital, Singapore, Singapore

**Keywords:** Intrabiliary, Intraductal, Hepatocellular carcinoma, Endoscopic retrograde cholangiopancreatography, Case report

## Abstract

**Background:**

Hepatocellular carcinoma with biliary ductal invasion is rare and associated with a significantly lower survival rate.

**Case presentation:**

We present an unusual case of a patient with hepatocellular carcinoma and biliary invasion, who had his diagnosis confirmed by histological analysis from tissue extracted by endoscopic retrograde cholangiopancreatography. An 87-year-old male presented with a 1-day history of right upper quadrant pain and jaundice. His past medical history included recurrent gallstone cholangitis and a previous cholecystectomy. An abdominal CT demonstrated a dilated intrahepatic biliary tree with left proximal intrahepatic hyperdensities, as well as a 3 cm hepatocellular carcinoma. He was initially suspected to have concurrent gallstone cholangitis and a newly diagnosed hepatocellular carcinoma. Endoscopic retrograde cholangiopancreatography and balloon trawling of the intraductal lesions extracted necrotic tumour-like tissue which was histologically consistent with hepatocellular carcinoma. The extraction of the intra-biliary portion of HCC resulted in complete resolution of his jaundice, enabling further treatment with nivolumab, which would not have been possible if the obstruction was not cleared. The patient is currently well and has completed his 6th cycle of nivolumab.

**Conclusion:**

Obstructive jaundice is an uncommon presentation for patients with HCC. it is key for clinicians to be aware of the possibility of intrabiliary invasion in order obtain an early diagnosis and to reduce any delay in treatment.

## Background

Hepatocellular carcinoma (HCC) is the most common primary malignant hepatic neoplasm. However, it only rarely shows biliary ductal invasion, with a reported incidence of 1–9% [1, 2]. We discuss an unusual presentation of a patient with obstructive jaundice secondary to HCC with intrabiliary invasion, who had his diagnosis confirmed from tissue that was retrieved by endoscopic retrograde cholangiopancreatography (ERCP). These patients typically have a poor prognosis. Therefore, it is key for clinicians to be aware of this entity in order obtain an early diagnosis and to reduce any delay in treatment.

## Case presentation

An 87-year-old Chinese male presented with right upper quadrant pain and obstructive jaundice for one day. He had no fever, nausea, or vomiting. He had no history of alcohol use. His medical history was significant for hyperlipidemia, diabetes mellitus, recurrent gallstone cholangitis, and a previous cholecystectomy 20 years ago. Of note, he had presented with gallstone cholangitis 4 months prior, during which an ERCP had been performed with successful balloon trawling of a 14 mm gallstone from the distal common bile duct. Physical examination revealed scleral icterus and mild right hypochondrial tenderness. His laboratory workup was notable for raised total bilirubin of 81 μmol/L, a low serum albumin of 33 g/L, thrombocytopenia, and an elevated serum alphafetoprotein level of 611μg/L. He had no leukocytosis, and his INR was normal.

**Fig. 1:**
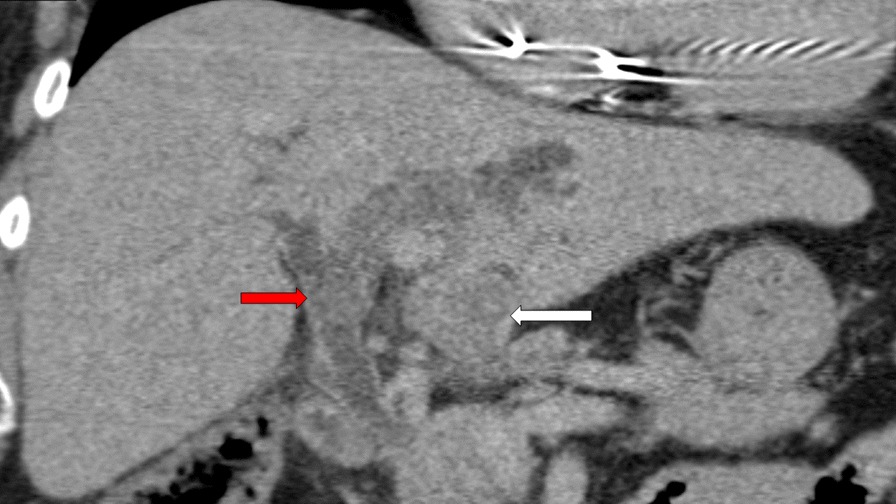
Multiphasic computed tomography scan showed a 3.1 cm lesion in segment 3 of the liver with arterial enhancement and delayed washout suspicious for HCC (white arrow), and a dilated intra- and extra-hepatic biliary tree with thickening and enhancement of the ductal wall as well as a hyperdensity in the proximal left hepatic duct (red arrow) suggestive of choledocholithiasis

A computed tomography multiphasic scan of his abdomen showed a dilated intra- and extra-hepatic biliary tree with thickening and enhancement of the ductal wall as well as hyperdensities within the proximal left hepatic duct and common hepatic duct suggestive of choledocholithiasis. It also showed a 3 cm segment 3 lesion with faint arterial enhancement and subsequent washout on the delayed phase suggestive of HCC (Fig. 1). Given his history of recurrent gallstone cholangitis, the initial impression was that of gallstone cholangitis and a possible newly diagnosed HCC. However, the biochemical profile and radiological appearance of his liver were not in keeping with cirrhosis. He was a non-drinker and his hepatitis B and C serology were negative. This patient was obese with metabolic risk factors such as diabetes and hypertension, suggesting that non-alcoholic steatohepatitis was the likely aetiology for his HCC.

**Fig. 2:**
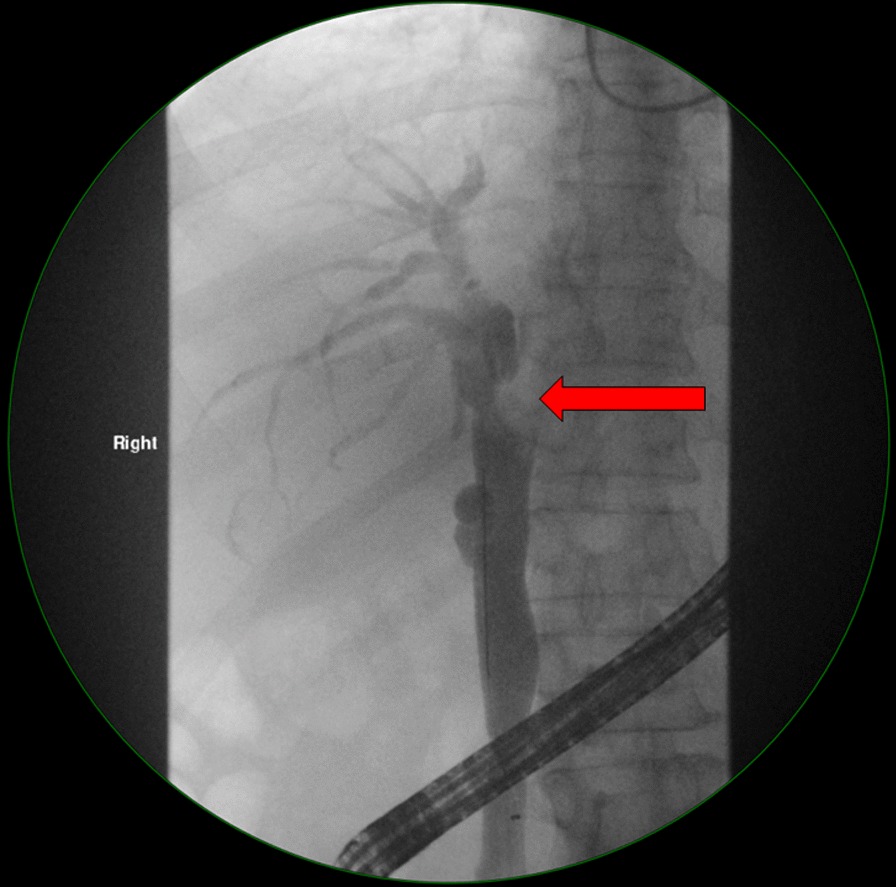
Cholangiogram demonstrating a large filling defect (red arrow) obstructing the distal left hepatic duct

**Fig. 3:**
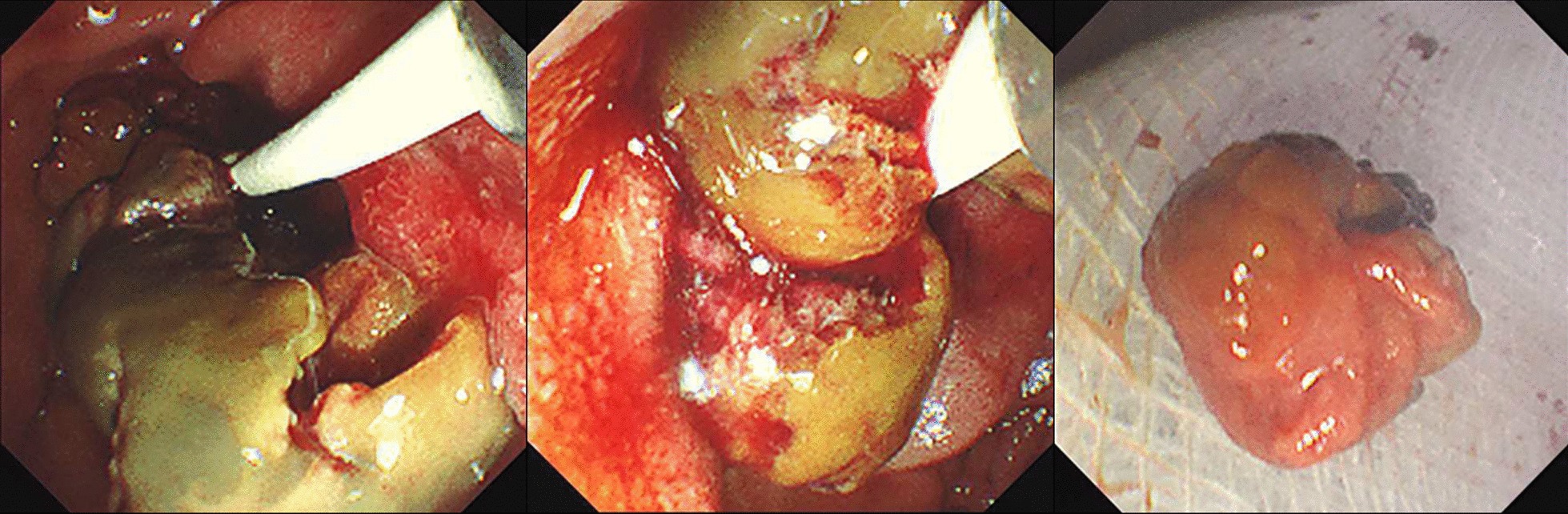
Extraction of necrotic tumour-like tissue via balloon trawling during ERCP

**Fig. 4:**
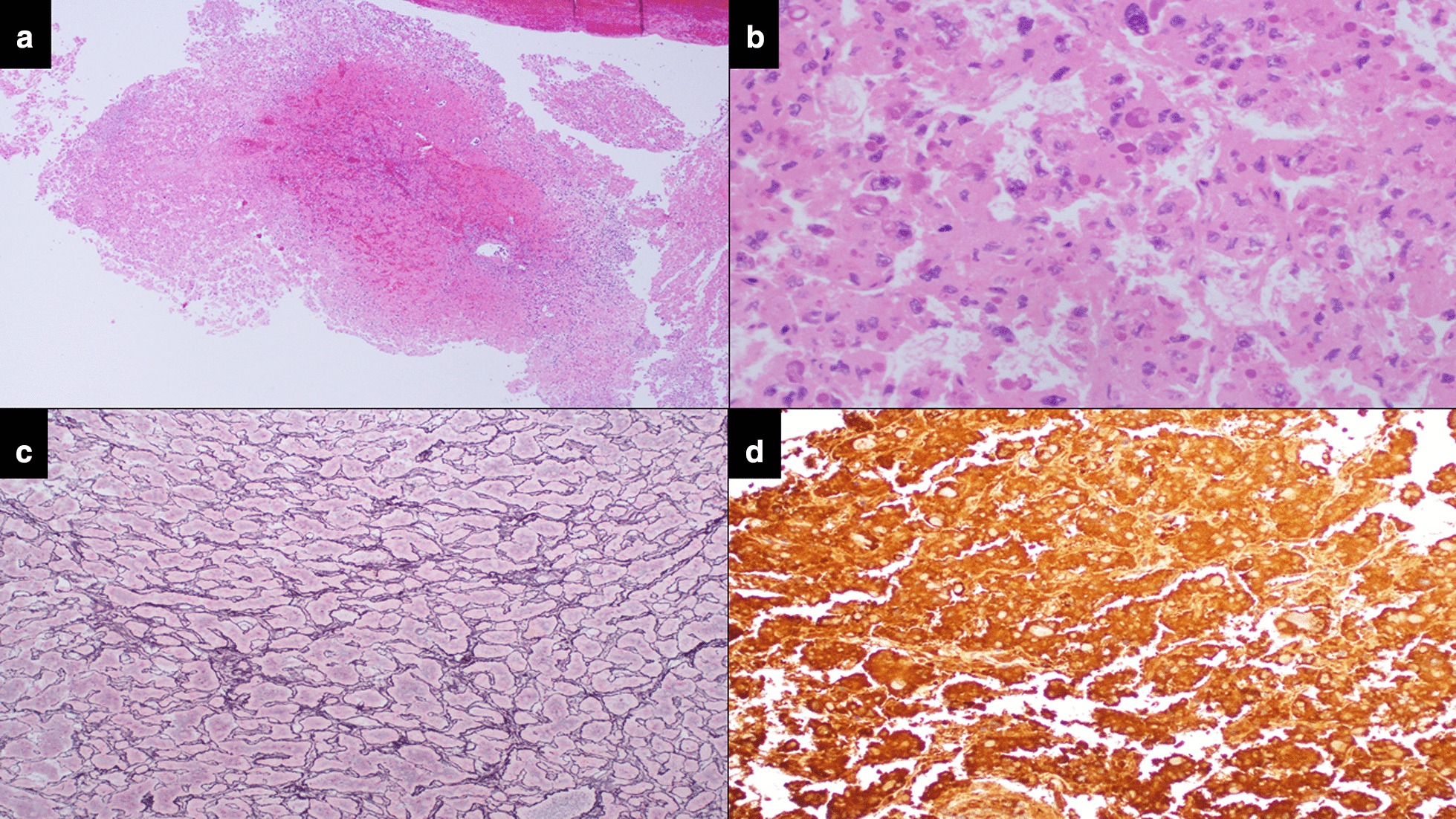
Histologic examination of the specimen. **a** Infarcted and haemorrhagic pieces of tissue with ghost outlines of necrotic cells (hematoxylin & eosin stain, 40x). **b** Retained morphology showing irregular pleomorphic nuclei with prominent nucleoli and numerous hyaline globules (hematoxylin & eosin stain, 400x). **c** Trabecular architecture (reticulin stain, 200x). **d** Diffuse staining of tumour cells on HepPar-1 immunostain. (HepPar-1 immunostain; 200x)

An ERCP was performed which demonstrated a dilated left intrahepatic duct with amorphous filling defects obstructing its distal end (Fig. 2). Balloon trawling during the ERCP managed to extract the obstructing intraductal lesions, which appeared to be necrotic tumour-like tissue (Fig. 3). These lesions were later retrieved with a Roth Net. No prophylactic stent was placed as the obstruction had resolved. Histology showed necrotic tumour tissue, with the residual partially viable tumour cells showing morphology (trabecular architecture and hyaline globules) as well as immunoreactivity (diffusely positive staining with HepPar, Arginase, and CAM5.2) that was compatible with HCC (Fig. 4).

After discussion at a multidisciplinary tumour board meeting, the options of surgical resection and systemic therapy were both offered to the patient. However, the patient opted for systemic therapy in view of his age and personal preferences, understanding that this was a palliative option. The extraction of the intra-biliary portion of HCC resulted in complete resolution of his jaundice, allowing initiation of systemic therapy. Nivolumab was eventually started after discussion with the patient and his family. A repeat computed tomography scan after completing six cycles of nivolumab showed partial response, and the patient remains well and free of jaundice.

## Discussion and conclusions

HCC only rarely leads to bile duct invasion, with a reported incidence of 1% [1]. These patients may present with obstructive jaundice as the initial complaint. There are several mechanisms through which HCC may cause obstructive jaundice: a direct invasion of the HCC into the biliary tree; HCC rupture within the biliary tree; a portal thrombus invading the biliary tree; tumour haemorrhage leading to blood clots and haemobilia; or extrinsic biliary compression from tumour or malignant lymphadenopathy [2].

Despite advancements in hepatobiliary imaging, the diagnosis of an intrabiliary HCC remains challenging, and is often confused with other causes of intrabiliary filling defects, such as choledocholithiasis, biliary cystadenomas and cholangiocarcinomas [3]. Intrabiliary HCC has been described to exhibit the same characteristic imaging features as HCC of arterial phase hyperenhancement and delayed washout on the portal venous phase [4]. However, there are no imaging features to reliably differentiate intrabiliary HCC from the other causes of intrabiliary filling defects. Thus, diagnosis is often determined by histology [5].

Obstructive jaundice is typically a late clinical manifestation of intrabiliary HCC. Prognosis is generally poor, and is closely related to the stage of disease, location of tumour and degree of tumour extension. The mean survival rate ranges from 2 to 4.5 months amongst several studies [1, 6, 7]. Cholangitis secondary to tumour obstruction is the most common cause of death in these patients.

We initially suspected choledocholithiasis as the cause of the obstructive jaundice in this case, given his significant history of recurrent gallstone cholangitis with a recent episode prior to presentation. However, the diagnosis of HCC was subsequently clinched when the tumour tissue was trawled out during an ERCP. To our knowledge, there has only been one other case report in the literature where HCC was also diagnosed from tissue removed during an ERCP [8]. In cases of HCC with intrabiliary invasion, surgical resection is the ideal treatment as it can result in resolution of jaundice and even long-term survival [2]. A study by Zeng et al. included 37 patients with bile duct tumour thrombus who had undergone liver resection between 2005 to 2012 and reported an overall survival of 64.2% at 1 year and 24.3% at 3 years. HCC patients with jaundice secondary to biliary obstruction should be managed differently from those with jaundice secondary to hepatic insufficiency, as curative surgical resection may still be an option in the former [9].

In conclusion, the diagnosis of intrabiliary invasion of HCC is rare, but needs to be considered when intraductal filling defects are noted on imaging, especially in the context of underlying liver disease and cirrhosis.

## Data Availability

Data and material used will be available from the corresponding author.

## Abbreviations

HCC: Hepatocellular carcinoma
ERCP: Endoscopic retrograde cholangiopancreatography
CT: Computed tomography
MRCP: Magnetic resonance cholangiopancreatography
H&E: Hematoxylin & eosin

## References

[1] Kojiro M, Kawabata K, Kawano Y, Shirai F, Takemoto N, Nakashima T (1982). Hepatocellular carcinoma presenting as intrabile duct tumor growth: a clinicopathologic study of 24 cases. Cancer..

[2] Qin LX, Tang ZY (2003). Hepatocellular carcinoma with obstructive jaundice: diagnosis, treatment and prognosis. World J Gastroenterol.

[3] Kim AY, Jeong WK (2016). Intraductal malignant tumors in the liver mimicking cholangiocarcinoma: imaging features for differential diagnosis. Clin Mol Hepatol.

[4] Diaz-Ruiz MJ, Falcó J, Martin J, Bella RM, Carrasco M, Tortajada L (2000). Hepatocellular carcinoma presenting as portal thrombosis with intrabiliary growth: US and MR findings. Abdom Imaging.

[5] Tseng JH, Hung CF, Ng KK, Wan YL, Yeh TS, Chiu CT (2001). Icteric-type hepatoma: magnetic resonance imaging and magnetic resonance cholangiographic features. Abdom Imaging.

[6] Lau WY, Leung KL, Leung TW, Ho S, Chan M, Liew CK (1995). Obstructive jaundice secondary to hepatocellular carcinoma. Surg Oncol.

[7] Huang GT, Sheu JC, Lee HS, Lai MY, Wang TH, Chen DS (1998). Icteric type hepatocellular carcinoma: revisited 20 years later. J Gastroenterol.

[8] van Dinter TG, Schmidt JF, Tarnasky PR (2011). Obstructive jaundice caused by intraductal hepatocellular carcinoma. Clin Gastroenterol Hepatol.

[9] Zeng H, Xu LB, Wen JM, Zhang R, Zhu MS, Shi XD (2015). Hepatocellular carcinoma with bile duct tumor thrombus: a clinicopathological analysis of factors predictive of recurrence and outcome after surgery. Medicine..

